# Sociodemographic disparities in corticolimbic structures

**DOI:** 10.1371/journal.pone.0216338

**Published:** 2019-05-09

**Authors:** Danielle Shaked, Zachary B. Millman, Danielle L. Beatty Moody, William F. Rosenberger, Hui Shao, Leslie I. Katzel, Christos Davatzikos, Rao P. Gullapalli, Stephen L. Seliger, Guray Erus, Michele K. Evans, Alan B. Zonderman, Shari R. Waldstein

**Affiliations:** 1 Department of Psychology, University of Maryland, Baltimore County, Baltimore, Maryland, United States of America; 2 Laboratory of Epidemiology and Population Sciences, National Institute on Aging Intramural Research Program, Baltimore, Maryland, United States of America; 3 Department of Statistics, George Mason University, Fairfax, Virginia, United States of America; 4 Geriatric Research Education and Clinical Center, Baltimore VA Medical Center, Baltimore, Maryland, United States of America; 5 Division of Gerontology & Geriatric Medicine, Department of Medicine, University of Maryland School of Medicine, Baltimore, Maryland, United States of America; 6 Section for Biomedical Image Analysis, University of Pennsylvania, Philadelphia, Pennsylvania, United States of America; 7 Department of Diagnostic Radiology, University of Maryland School of Medicine, Baltimore, Maryland, United States of America; 8 Division of Nephrology, Department of Medicine, University of Maryland School of Medicine, Baltimore, Maryland, United States of America; University of Colorado Denver, UNITED STATES

## Abstract

This study sought to examine the interactive relations of socioeconomic status and race to corticolimbic regions that may play a key role in translating stress to the poor health outcomes overrepresented among those of lower socioeconomic status and African American race. Participants were 200 community-dwelling, self-identified African American and White adults from the Healthy Aging in Neighborhoods of Diversity across the Life Span SCAN study. Brain volumes were derived using T1-weighted MP-RAGE images. Socioeconomic status by race interactions were observed for right medial prefrontal cortex (B = .26, *p* = .014), left medial prefrontal cortex (B = .26, *p* = .017), left orbital prefrontal cortex (B = .22, *p* = .037), and left anterior cingulate cortex (B = .27, *p =* .018), wherein higher socioeconomic status Whites had greater volumes than all other groups. Additionally, higher versus lower socioeconomic status persons had greater right and left hippocampal (B = -.15, *p* = .030; B = -.19, *p* = .004, respectively) and amygdalar (B = -.17, *p* = .015; B = -.21; *p* = .002, respectively) volumes. Whites had greater right and left hippocampal (B = -.17, *p* = .012; B = -.20, *p* = .003, respectively), right orbital prefrontal cortex (B = -.34, *p* < 0.001), and right anterior cingulate cortex (B = -.18, *p* = 0.011) volumes than African Americans. Among many factors, the higher levels of lifetime chronic stress associated with lower socioeconomic status and African American race may adversely affect corticolimbic circuitry. These relations may help explain race- and socioeconomic status-related disparities in adverse health outcomes.

## Introduction

There is a well-known association between lower socioeconomic status (SES) and a range of adverse health outcomes. Disadvantaged socioeconomic positions are associated with higher morbidity and mortality rates across many chronic diseases, including cardiovascular [1], cerebrovascular [2], and chronic kidney [3] disease. Although the mechanisms accounting for the SES-health relation are not fully understood, one biologically plausible pathway is the long-term activation of the neural stress response. During acute stress, the brain is in constant communication with the autonomic, neuroendocrine, and immune systems to maintain homeostasis [4]. Eventually, however, long-term stressors such as lower socioeconomic environments may lead to wear-and-tear on the body and brain, frequently resulting in ill health, perhaps in part as a result of repeated or prolonged activations of these psychophysiological systems [5].

**Fig 1:**
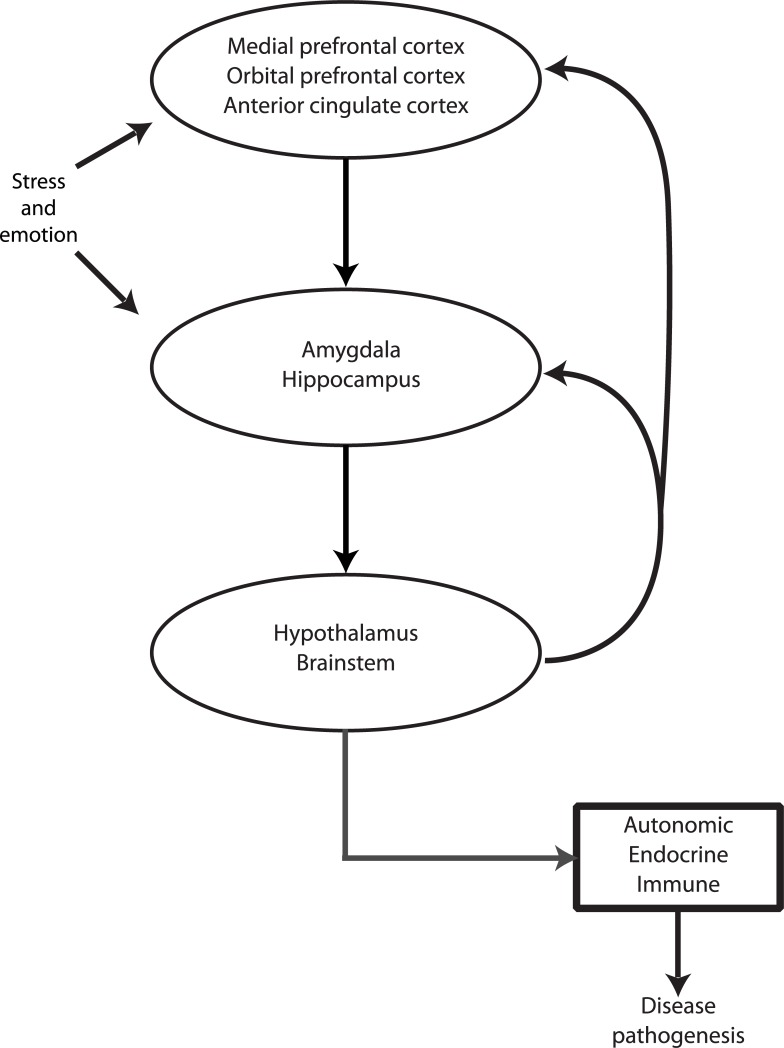
Simplified Heuristic of the corticolimbic circuit and its potential link to disease pathogenesis. It is posited that the translation of stress and emotion to disease outcomes involves bidirectional communication among several cortical and subcortical brain regions known to influence autonomic, endocrine, and immune system function via neural and hormonal mechanisms. Higher-order cortical regions involved in emotion regulation and stress perception, including the medial PFC, orbital PFC, and ACC, help modulate the function of subcortical regions, such as the hippocampus and amygdala, that are involved in emotion and stress processing (e.g., rapidly processing the emotional salience of an event). These subcortical regions, in turn, modulate lower-order brain structures such as the hypothalamus and brainstem, which are directly responsible for the release of catecholamines and glucocorticoids by peripheral systems (e.g., autonomic nervous system, the HPA axis). Repeated and/or prolonged activation of these peripheral systems may ultimately impair the structure and function of these corticolimbic regions. A dysregulated corticolimibic circuit may ultimately lead to disease states via repeated and/or chronic psychophysiological stress responses. Please refer to Fig 2 for visualization of relevant brain structures.

Various neural pathways may participate in the translation of stressful life circumstances to poor health [5, 6]. Research suggests that one pathway involves chronic activation of the corticolimbic circuitry involved in emotion and stress reactivity [4]. In that regard, cortical structures, such as the anterior cingulate cortex (ACC) and the medial and orbital subregions of the prefrontal cortex (PFC), are crucial for detecting and appraising stressful environmental stimuli, generating and regulating an emotional response, and bi-directionally communicating with peripheral systems (e.g., autonomic, neuroendocrine, and immune) [4]. Notably, communication of the PFC with these physiological systems is mediated by multiple subcortical structures, particularly the amygdala and hippocampus [7]. These structures are core components of the corticolimbic circuit and are central to regulating the physiological stress responses produced by lower-order brain regions, such as the hypothalamus and brainstem [5]. During conditions of psychological stress, these lower-order brain regions stimulate increased secretion of catecholamines (e.g., noradrenaline and dopamine) and glucocorticoids (e.g., cortisol), which can ultimately impair cortical and subcortical structure and function [8–10]. This heightened neurotransmitter/hormone release triggers a cycle wherein the cortical structures eventually can no longer effectively modulate the subcortical structures, thus leading to dysregulation of the emotion/stress response and, ultimately, disease states (Fig 1). Consistent with other researchers (e.g., [4]), we hypothesize that the impaired structure and function of these corticolimbic regions (such as reduced volume) may impair efficient modulation of lower-order brain areas, such as the hypothalamus and the brain stem. Relatedly, animal and human studies have demonstrated relations between stress exposure and morphological changes in a number of corticolimbic brain regions, including the ACC (e.g., [11]), orbital (e.g., [12]) and medial PFC (e.g., [9, 11]), hippocampus (e.g., [13–15]), and amygdala (e.g., [14, 16, 17]). In these studies, greater stress exposure is consistently related to smaller gray matter volume. While the directionality of these effects is challenging to delineate, the impact is likely bidirectional given the literature showing a) the direct impact of stress on brain plasticity (e.g., [13, 18]) and b) increased vulnerability of inefficient stress processing as a result of relatively smaller corticolimbic brain structures [19–21]. Moreover, it is thought that stress is a developing process that influences health throughout the lifespan, involving complex interactions between psychological, physiological, cultural, environmental, and other biopsychosocial factors [22].

In addition to experiencing a greater number of acute stressors, daily life for individuals with low SES is generally perceived as less safe relative to those with higher SES [23]. The Generalized Unsafety Theory of Stress [23] states that when the brain perceives the environment to be unsafe the stress response is chronically enhanced, thus contributing to a dysregulated corticolimbic circuit (Fig 1). This is consistent with the literature showing that those with perceived low SES, on average, have increased sympathetic nervous system activity [24] and reduced volumes of the ventral ACC [25], an area thought to be involved in regulating parasympathetic responses and thus important for modulating the fight or flight (i.e., stress) response [26]. As lower SES may be related to higher levels of stress and generalized unsafety, as well as adverse changes to corticolimbic brain regions and associated physiological systems, it is possible that socioeconomic stress translates to disease, in part, by overburdening cortical structures and, in turn, the subcortical regions they modulate.

Consistent with this hypothesis, a growing body of literature suggests that lower SES environments are associated with poorer structural and functional neuroanatomical outcomes (for reviews see [5, 27, 28]). Several studies investigating these relations in children found that lower levels of subjective and objective SES were related to functional and structural changes in various brain regions involved in stress perception/physiology and emotion processing/regulation, including the hippocampus, amygdala, ACC, and PFC [29–33]. For instance, an fMRI study showed that in a complex stimulus-response learning task that elicits the medial PFC, children from lower SES backgrounds performed more poorly than children from higher SES backgrounds [31]. Concurrently, the low SES group showed greater activation of the medial PFC during the task, possibly reflecting less efficient processing. Additionally, structural imaging studies have shown that children from lower SES backgrounds have smaller volumes in the hippocampus and amygdala [29, 32], and that in adults, lower subjective SES is related to lesser ventral ACC [25].

In addition to regional differences, researchers have also examined variability in global brain outcomes among SES groups. In a large sample of midlife adults, a recent study by Gianaros and colleagues (2017) found that low SES was related to lower overall cortical gray matter volume, and that increased cardiometabolic risk and dysregulated diurnal cortisol mediated this association. Although not all investigations corroborate the relation between SES and global brain volume [34, 35], similar findings between SES and global brain volume were found in a portion of the current study’s sample [36].

The mediating effect of cortisol on the relation between SES and gray matter [37] suggests a role of stress neurobiology in the relation between SES and brain morphology. Given evidence that SES is related to both global and regional brain volumes, perhaps stress-related factors contribute to both. Further evidence of this is suggested by studies finding that childhood maltreatment is associated with perturbed neurodevelopment [38–40]. Other mediating mechanisms, however, are also suggested by the literature. For instance, cardiometabolic risk has been shown to mediate the SES-brain relation [37], possibly as a function of environmental disparities such as access to nutritional food and safe green space for physical activity. Engagement of health behaviors, the home and school environment in which children develop, mental and physical health, access to care and material resources, linguistic environment, and neurotoxic exposure, may also be among the many multilevel influences contributing to neuroanatomical disparities among SES groups (for reviews see [5, 28]).

The associations between SES and brain volume may further vary by self-identified race. Research suggests that earlier health deterioration among African Americans (AAs) compared to Whites is related to the physiological “wear-and-tear” resulting from disproportionate levels of stress exposure and perceptions of generalized unsafety, due to environmental adversities, such as discrimination [41–43]. Perhaps as a function of these contextual considerations, prior research suggests that, on average, AAs may not benefit (as much as Whites) from higher socioeconomic positions with respect to health, a possibility termed as the “diminishing returns hypothesis” [44]. For example, our lab has recently shown that compared to Whites with high SES, AAs with high SES had lower overall brain volume; Whites with low SES, however, presented similarly to AAs with low and high SES [36]. These findings parallel studies demonstrating race- and SES-related disparities in physical health [45, 46]. Given these findings, it is possible that higher SES is not as protective for AAs as it is for Whites with respect to stress-relevant neuroanatomical regions. Considering the brain’s presumed role in mediating chronic health outcomes, this possible disadvantage may help explain the preponderance of health disparities across AAs and Whites.

Here, we examined the potential interactive associations of SES and self-identified race to magnetic resonance imaging (MRI)-assessed regional gray matter volume of select corticolimbic structures, including the orbital and medial PFC and ACC (cortical structures), as well as the hippocampus and amygdala (subcortical structures). Given the literature demonstrating poorer structural neuroanatomical outcomes in individuals with lower SES and AAs, we hypothesized that Whites high in a composite SES index of poverty status and education would present with greater volumes in the examined brain regions compared to all other groups. While not assessed directly here, we hypothesized that a lifetime accumulation of biopsychosocial stressors experienced by individuals with lower SES and AAs may, in part, explain these potential relations. In exploratory analyses, we examined interactive relations of race with several individual SES indicators for each of the primary brain regions. To our knowledge, this is the first study to examine the synergistic effects of SES and race on stress-relevant brain regions in a socioeconomically diverse group of AA and White community-dwelling adults.

## Materials and methods

### Participants

Participants were 200 community-dwelling adults (mean age = 52 years; 44% male, 58% White, high SES = 51%) in the Healthy Aging in Neighborhoods of Diversity across the Life Span (HANDLS) study who had completed a visit during 2009–2013. HANDLS is an ongoing, 20-year longitudinal study aimed at understanding health disparities across a socioeconomically diverse group of AA and White adults residing in 13 pre-selected Baltimore neighborhoods [47]. The HANDLS study was approved by the Institutional Review Board (IRB) of the National institute of Environmental Health Services, National Institutes of Health. The current participants were recruited from HANDLS to take part in HANDLS SCAN, an ancillary imaging study to HANDLS [36].

HANDLS study exclusions were 1) outside of the age range 30–64 years; 2) currently pregnant; 3) within six months of receiving chemotherapy, radiation, or biological treatments for cancer; 4) diagnosed with AIDS; 5) unable to provide informed consent due to mental incapacity resulting from drug or alcohol intoxication, severe developmental disability, or dementia; 6) unable to complete at least five of the nine tests given on the Mobile Medical Research Vehicle; 7) without a verifiable address or valid government issued identification at time of consent. HANDLS SCAN participants had the following additional exclusions: history of dementia, stroke or transient ischemic attack; history of carotid endarterectomy; MRI contraindications (e.g., claustrophobia, indwelling ferromagnetic material); diagnosis of a terminal illness; HIV positive status; or other neurological disorders (e.g., multiple sclerosis, Parkinson’s disease). Two hundred participants had complete data necessary for this study.

Eligible HANDLS participants were approached during their examination and invited to participate in HANDLS SCAN. If interested, participants were contacted, administered an MRI eligibility screener, and scheduled by a research coordinator. They provided both written informed and HIPAA consent approved by the IRBs at the University of Maryland, Baltimore and the University of Maryland, Baltimore County for HANDLS SCAN. Participants were then examined by a physician at the University of Maryland General Clinical Research Center to review current medications, re-administer the MRI eligibility checklist, assess whether there were any contraindications to the MRI testing, and provide a brief medical evaluation to identify any acute medical problems since their last HANDLS visit. Finally, they proceeded to the Department of Diagnostic Radiology & Nuclear Medicine at the University of Maryland School of Medicine to undergo MRI testing.

### Measures

#### Demographic variables

The SES category was computed using years of education and poverty status. Education was dichotomized into above or below median education (0 = 12 years or above or received a GED; 1 = < 12 years). Household income was dichotomized into above or below 125% of the 2004 federal poverty line adjusted for household size (e.g., $23,562 per year for a family of four), coded as 0 = above poverty line and 1 = below poverty line. SES was dichotomized into high and low SES based on these two variables. Low SES was defined as having low education (< 12 years), being below the poverty line, or both. Participants were classified as high SES if they were in both the high education group and above the poverty line. Self-identified race was dichotomized into 0 = White and 1 = AA. Sex (0 = female; 1 = male) and age (in years) were also assessed.

#### Magnetic resonance imaging

Participants underwent MRI using a Siemens Tim-Trio 3.0 Tesla scanner. Volumetric measurements for anatomical regions were calculated using magnetization prepared rapid gradient echo (MP-RAGE). The T1-weighted MP-RAGE images covered the entire brain in the sagittal plane at 1.2 mm thickness for a total of 160 slices (TR/TE/TI = 2300/2.9/900 ms; FOV 25.6cm). For comparison purposes, these images were converted from sagittal to axial sections.

In-house techniques developed by the Section for Biomedical Image Analysis at the University of Pennsylvania were used to preprocess structural MRI scans. First, skull-stripping was applied to remove extra-cranial material on the T1-weighted images using, a multi-atlas registration based method that requires minimal manual correction [48]. Bias correction was performed using the multiplicative intrinsic component optimization (MICO) method [49]. Preprocessed images were segmented into a set of anatomical regions of interest (ROIs) using a new multi-atlas label fusion method, MUlti-atlas region Segmentation utilizing Ensembles (MUSE), which has achieved state-of-the-art accuracy in segmentation of brain structures in an independent comparative evaluation [50]. This sophisticated image segmentation method integrates a broad ensemble of labeled templates through the use of several warping algorithms, regularization parameters, and atlases [50]. This atlas partitions over 100 ROIs, although it does not include all brain regions (e.g., hypothalamus). The creators of this method say: “this approach outperforms methods that are based on single parameter sets and registration algorithms, and can therefore provide a foundation for robust segmentation (p. 12,[40])” Volumetric measurements were calculated for each individual ROI.

**Fig 2:**
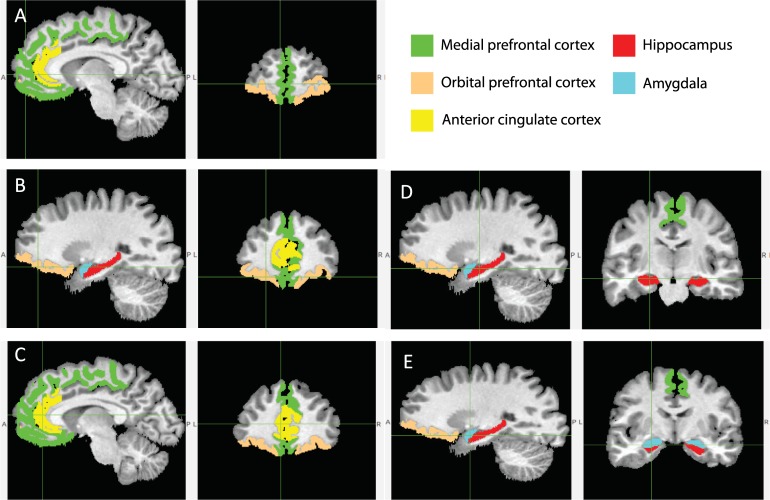
**Cortical (A-C) and Subcortical (D-E) Regions of Interest.** Regions are delineated by the cross, and the colors are consistent throughout the figure. Regions are depicted on both a sagittal (left) and coronal (right) plane.

The primary ROIs examined here were gray matter volumes of the right (R) and left (L) medial and orbital regions of the PFC, ACC, hippocampus, and amygdala (Fig 2). Given the medial location of the medial PFC and ACC, supplementary analyses were run looking at the total volume of these structures, without laterality distinctions. Occipital pole (encompassing much of the primary visual cortex) was examined as a supplementary control analysis. The ACC, hippocampus and amygdala were pre-specified. As shown in Fig 2, our delineation of the ACC includes the ventral portion of the ACC (i.e., perigenual and subgenual ACC) and a portion of the dorsal ACC (i.e., mid cingulate ACC). The medial PFC was created by summing the gyrus rectus, medial frontal cortex, precentral gyrus medial segment, superior frontal gyrus medial segment, subcallosal area, and the supplementary motor area. While there are important functional subdivisions of the medial PFC (e.g., dorsomedial PFC and ventromedial PFC) we chose to examine the entire medial PFC given the literature suggesting that the entire region is important for stress and emotion processing [6, 51]. The orbital PFC was created by summing the anterior, lateral, medial, and posterior orbital gyri. The R and L portions of the occipital pole, medial PFC, and ACC were summed together.

The ROIs were not corrected for intracranial volume (ICV) for the following reasons: a) main differences in ICV are primarily due to sex and age [52], which were covariates in all of our analyses, b) we were interested in the actual volumes of the ROIs, as opposed to volumes relative to the entire brain, c) given that ICV is highly correlated with most ROIs, parsing out ICV would remove much of the ROIs’ variance, and d) there is known to be bias in ICV estimation [53].

#### Data analysis

Multiple regression analyses (SPSS 22.0 and SAS 9.4) examined the interactions of SES and race and their first-order associations with each R and L ROI. All analyses were adjusted for age and sex. If a brain region did not reveal a significant interaction, backwards elimination was used to estimate main effects. Following this procedure, we first included the interaction term in the regression, and then stepwise removed any non-significant higher order interaction to test if there would be a model with significant main effect predictors.

Utilizing the same multiple regression models, a supplemental control analysis was conducted on the occipital pole. The following exploratory analyses were conducted: 1) Total medial PFC and ACC were examined as outcome variables and 2) interactive relations of race with several individual SES indicators including continuous education, dichotomous education, and poverty status were examined for each of the primary ROIs. Findings were considered statistically significant if alpha (*p*) was less than .05. To be more conservative, statistically significant effects were considered meaningful if the corresponding standardized regression coefficient (B) was greater than .20 [54]. Significant interactions were probed using PROCESS Model 1 [55].

## Results

### Preliminary analyses

Post hoc power analysis was conducted using G*Power 3.1.3 statistical software. For medium effects to be detected (*f*^2^ estimate of 0.20), using an *a* = 0.05, a power of .99 was achieved, demonstrating that adequate power can be achieved from these analyses in order to detect a moderate effect in this sample. Regression diagnostics indicated an adequate fit to the data [56]. Table 1 shows demographic data for the overall sample, and by race and SES. As demonstrated, there were significant group differences. For race, AAs were significantly younger (*t*(198) = 2.34, *p* = .020) and more likely to be living in poverty (*F*(1, 198) = 8.44, *p* = .004). For SES, the low SES group was significantly younger (*t*(198) = 3.05, *p* = .003) and predominantly female (*F*(1, 198) = 5.34, *p* = .022).

**Table 1:** Demographic characteristics in the overall sample and across race and SES.

Variable	Overall (N = 200)	White (n = 117)	AA (n = 83)	*p*^1^	Low SES (n = 99)	High SES (n = 101)	*p*^2^
Age in years, M (SD)	51.48 (9.12)	52.74 (8.41)	49.71 (9.82)	.020	49.54 (2.51)	53.39 (9.34)	.003
Years of Education, M (SD)	12.35 (2.70)	12.34 (3.08)	12.36 (2.06)	.960	10.89 (2.51)	13.78 (2.03)	< .001
Low Education (%)	25.50	29.10	20.50	.172	51.50	0.00	-
Below Poverty (%)	35.50	27.40	47.00	.004	71.70	0.00	-
Female (%)	55.50	53.00	59.00	.399	63.60	47.50	.022
AA (%)	41.50	-	-	-	48.50	34.70	.047
Low SES (%)	49.50	43.60	57.80	.047	-	-	-

AA = African Americans; SES = socioeconomic status; M = mean; SD = standard deviation.

*p*^1^-value for the difference between African Americans and Whites; *p*^2^-value for the difference between high and low SES; independent samples t-tests were used for continuous variables (all equal variances assumed) and one-way ANOVAs were used for categorical variables.

### Analyses of the subcortical regions

No significant interactions were noted for the subcortical regions measured. Several main effects, however, were found (Table 2). After removing the non-significant interaction term from the regression, a main effect of race was found for the R hippocampus (B = -.17; *p* = .012), L hippocampus (B = -.20; *p* = .003), and was marginally significant for the L amygdala (B = -.13; *p* = .052), wherein Whites had greater volumes than AAs. A main effect of SES was found for the R hippocampus (B = -.15; *p* = .030), L hippocampus (B = -.19; *p* = .004), R amygdala (B = -.17; *p* = .015), and L amygdala (B = -.21; *p* = .002), wherein higher SES individuals had greater volumes. As noted, only the L amygdala superseded the B = .20 significance cutoff.

**Table 2:** Multiple regression analyses: Interaction of SES and race on subcortical gray matter volumes.

Variable (All Ns = 200)		*F*	*R*^*2*^	*p*	B	SE	*sr*^*2*^
R Hippocampus		9.92	.20	< .001			
	Age			.680	-.03	.07	< .001
	Sex			< .001	.35	.07	.12
	Race			.012	-.24	.09	.03
	SES			.019	-.20	.09	.02
	Race × SES			.289	.12	.11	.01
L Hippocampus		12.05	.24	< .001			
	Age			.154	-.09	.07	< .01
	Sex			< .001	.35	.06	.12
	Race			.002	-.29	.09	.04
	SES			.002	-.27	.08	.04
	Race × SES			.144	.16	.11	.01
R Amygdala		8.70	.18	< .001			
	Age			.509	-.05	.07	< .01
	Sex			< .001	.36	.07	.13
	Race			.437	-.07	.10	< .01
	SES			.004	-.25	.07	.03
	Race × SES			.127	.18	.12	.01
L Amygdala		12.71	.25	< .001			
	Age			.409	-.05	.07	< .01
	Sex			< .001	.38	.06	.14
	Race			.012	-.23	.09	.03
	SES			.001	-.29	.08	.05
	Race × SES			.104	.18	.11	.01

R = Right; L = Left; B = standardized regression coefficients; SE =

standard error; *sr*^*2*^ = semipartial correlation squared.

### Analyses of the cortical regions

**Fig 3:**
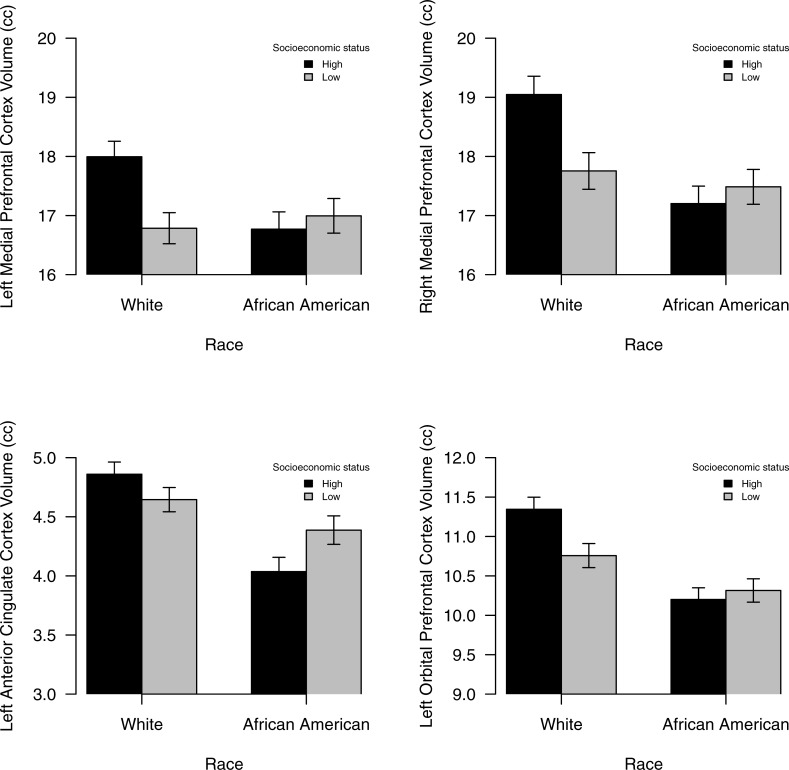
Graphical representation of significant SES by race interactions on various cortical brain regions. For all the structures depicted, high SES Whites had greater brain volumes relative to all other groups. Error bars depict the standard errors.

Significant interactions of SES and race were noted for the R (conditional effect: *p* < .001) and L (conditional effect: *p* = .004) medial PFC, L orbital PFC (conditional effect: *p* < .001), and the L ACC (conditional effect: *p* < .001; all Bs > .20; Table 3). As Fig 3 demonstrates, in all significant cases, Whites with higher levels of SES had greater volumes as compared to all other groups. After removing the non-significant interaction term from the regression, race main effects were found for the R orbital PFC (B = -.34; *p* < .001) and the R ACC (B = -.18; *p* = .011), showing greater volume for Whites than AAs. SES and race interactions were also significant for total medial PFC (conditional effect: *p* < .001) and ACC (conditional effect: *p* < .001; all Bs > .20; S2 Table).

**Table 3:** Multiple regression analyses: Interaction of SES and race on cortical gray matter volumes.

Variable (All Ns = 200)		*F*	*R*^*2*^	*p*	B	SE	*sr*^*2*^
R Medial PFC		17.74	.31	< .001			
	Age			.001	-.20	.06	.04
	Sex			< .001	.41	.06	.16
	Race			< .001	-.38	.09	.07
	SES			.003	-.24	.08	.03
	Race × SES			.014	.26	.11	.02
L Medial PFC		15.99	.29	< .001			
	Age			.002	-.20	.06	.04
	Sex			< .001	.43	.06	.18
	Race			.002	-.27	.09	.04
	SES			.004	-.24	.08	.03
	Race × SES			.017	.26	.11	.02
R Orbital PFC		21.44	.36	< .001			
	Age			.330	-.06	.06	< .01
	Sex			< .001	.45	.06	.19
	Race			< .001	-.43	.08	.09
	SES			.054	-.15	.08	.01
	Race × SES			.107	.17	.10	.01
L Orbital PFC		18.44	.32	< .001			
	Age			.031	-.13	.06	.02
	Sex			< .001	.42	.06	.17
	Race			< .001	-.43	.09	.09
	SES			.023	-.18	.08	.02
	Race × SES			.037	.22	.10	.02
R ACC		4.69	.11	< .001			
	Age			.002	-.22	.07	.05
	Sex			.032	.15	.07	.02
	Race			.007	-.27	.10	.03
	SES			.199	-.12	.09	.01
	Race × SES			.193	.16	.12	.01
L ACC		9.90	.20	< .001			
	Age			.016	-.16	.07	.02
	Sex			.001	.22	.07	.05
	Race			< .001	-.49	.09	.12
	SES			.172	-.12	.09	.01
	Race × SES			.018	.27	.11	.02

R = Right; L = Left; PFC = prefrontal cortex; ACC = anterior cingulate

cortex; B = standardized regression coefficients; SE = standard error; *sr*^*2*^ =

semipartial correlation squared.

### Supplemental analyses

As displayed in S1 Table, findings with dichotomous education as the SES indicator yielded significant interactions for the L medial PFC (B = .18; *p =* .025) and L amygdala (B = .18; *p =* .034), although none of the corresponding Bs were greater than .20. Findings with poverty status as the SES indicator yielded significant interactions for the R medial PFC (B = .22; *p =* .034), R orbital PFC (B = .20; *p =* .041), and L ACC (B = .23; *p =* .040). No significant interactions were noted for continuous education. As detailed in S2 Table, supplemental analyses resulted in significant interactions for total medial PFC (B = .27, *p* = .011) and ACC (B = .34, *p* = .036). No significant interaction was noted for the occipital pole.

## Discussion

Socioeconomic disadvantage and self-identified AA race are risk factors for adverse health outcomes [43, 57–59]. One mechanistic pathway for these disparities could be that disproportionately higher levels of environmental stress exposure among many AAs and individuals with low SES have a negative effect on stress-sensitive brain regions putatively involved in disease pathogenesis. To better understand potential disease-related neural correlates of socioeconomic disadvantage and self-identified AA race, we examined the synergistic and independent relations of these demographic factors with stress-relevant brain regions, including the medial and orbital PFC, ACC, hippocampus, and amygdala. Consistent with our hypotheses, we found that compared to Whites with high SES, AAs with high and low SES and Whites with low SES were more likely to present with smaller volumes of the R and L medial PFC, L orbital PFC, and L ACC. These nuanced dynamics were not observed for the subcortical (and select cortical, i.e., R orbital PFC, R ACC) regions; in these regions, low SES and AA individuals, independently, displayed lesser volumes.

### Subcortical regions

The hippocampus and amygdala are core components of the corticolimbic circuit, particularly given their modulation of lower-order control of stress physiology (e.g., autonomic nervous system, hypothalamic-pituitary-adrenal [HPA] axis) [4]. The hippocampus and amygdala are involved in the identification of environmental threats and regulation of the physiological and behavioral responses to stressors [4]. We found these subcortical volumes were significantly lower in AAs relative to Whites, as well as in the low SES group relative to the high SES group. Given that smaller amygdala and hippocampus volumes are associated with stress-related physiological parameters [13, 17, 60], our findings suggest that AAs and individuals with low SES may on average have an increased vulnerability to an enhanced physiological stress response. It is worth noting that while these findings were all statistically significant, only the L amygdala demonstrated a substantial effect size (i.e., > .20) [54], and these results should therefore be interpreted with caution.

Our findings are consistent with prior studies demonstrating objective SES disparities in gray matter volumes of the amygdala [29] and hippocampus [15, 29, 32]. These results may be attributable, in part, to SES disparities in acute stress exposure, including a greater likelihood of exposure to family discord [61], neighborhood violence [61], as well as greater generalized unsafety and uncertainty [23]. Less is known about the racial disparities in these stress-relevant brain areas; disproportionate risks, such as discrimination, geographical segregation, and chronic stress experienced by many AAs may contribute [41, 43, 59].

### Cortical regions

In our study, high SES Whites presented with greater volumes in the R and L medial PFC, L orbital PFC, and L ACC relative to the other sociodemographic groups examined. Additionally, on average, Whites evidenced greater R orbital PFC and R ACC volumes compared to AA individuals. These cortical regions partly constitute the corticolimbic circuitry and are known to influence subcortical structures through top-down regulatory stress processes [5, 60]. Notably, these brain regions have a bidirectional influence; they modulate descending structures (e.g., the hippocampus) that in turn modulate stress-responsive physiological activity (e.g., the HPA axis), but can also be adversely impacted by subsequent prolonged output of substrates (e.g., cortisol; see Fig 1). Thus, inefficient functioning of these cortical regions can lead to excess levels of stress-related substrate release, which may result in structural changes to both the cortical regions themselves and the descending regions they modulate.

Only Whites displayed the expected SES-related advantages in cortical gray matter volume for the R and L medial PFC, L orbital PFC, and L ACC. For the R orbital PFC and R ACC Whites had higher volumes regardless of SES. This may in part reflect reduced exposure by Whites, particularly those of high SES, to lifelong environmental stressors that impact neurodevelopment. Specifically, the cerebrum develops in a caudal-to-rostral fashion, with anterior regions reaching full development later than posterior ones. Located in the most anterior portion of the cerebrum, the PFC has an extended ontogenetic trajectory, with development continuing well into the third decade of life [62]. As a result, the PFC is vulnerable to environmental insults for longer than most other brain regions [62]. Thus, for several of the brain regions considered, the concurrent socioeconomic advantage and racial privilege experienced by many Whites with high SES may make them disproportionately advantaged with regards to optimal cortical development, compared to all other groups considered in the present study.

One outcome of larger cortical volumes may be more efficient modulation of subcortical structures. While speculative, this may be reflected at the psychological level by production of more positive appraisals and use of efficient coping responses (and/or effective emotion regulation) during periods of acute and chronic stress [57]. These proactive strategies may, in turn, dampen the excessive stress-related substrate release from lower order brain regions. For instance, individuals with larger cortical volumes may have a greater ability to inhibit the amygdala’s instigation of prolonged excessive activity of the HPA and the cardiovascular stress response [60], thus preserving its structure and function, and alleviating the vicious cycle discussed previously. Therefore, the larger cortical volumes seen for Whites, particularly Whites with high SES, may provide this sociodemographic group with a greater ability to control the psychological and biological stress response.

Consistent with recent findings from our lab [36], high SES AAs did not benefit from their socioeconomic advantage with regards to total brain volume. As noted, stress-related factors have been shown to relate to global differences in brain volume in both children [39] and adults [37], although the findings examining SES and global brain outcomes are mixed [34–37]. Perhaps the higher rates of stress and generalized unsafety experienced by this sociodemographic group [23, 41] contribute to both global and region-specific disparities in brain structures. Given that these findings were not demonstrated in the occipital pole, an ROI that encompasses much of the primary visual cortex, perhaps the regional specificity is, at least in part, unique to stress-relevant cortical regions.

The lack of volumetric benefits seen in the high SES AA group is consistent with the “diminishing returns” hypothesis, which states that as levels of SES increase, AAs do not exhibit the same health improvements as their White counterparts [44]. Indeed the results of prior studies show that higher levels of education and income confer greater health benefits for Whites than AAs with regard to several systemic health outcomes such as subclinical atherosclerosis, cardiovascular disease, and inflammatory markers [45, 63, 64]. While the reasons for this discrepancy are largely unknown, a lifetime of environmental stressors (e.g., discrimination) experienced by AAs may contribute.

The results indicating significant SES and race interactions for the L, but not R, ACC and orbital PFC were unexpected. However, some studies have found that relative to the R ACC, the L ACC is more sensitive to chronic stress exposure [10] and glucocorticoid treatment [65]. Given these findings, some have hypothesized that regions of the left brain may be more vulnerable to the effects of corticosteroid levels, and even stress more generally [66]. These hypotheses might suggest that the privileged status of Whites with high SES fosters a healthier stress-response system in these individuals, ultimately leading to greater right, but mostly left, cortical volumes relative to other sociodemographic groups. This possibility, however, is speculative, and more research is needed to further delineate the lateralized contributions of cortical and subcortical regions involved in the neural stress response across sociodemographic groups.

Exploratory analyses showed that poverty status and education were differentially related to the brain regions, which is consistent with the literature noting that different socioeconomic factors may play different roles in brain plasticity [28]. For example, income and/or poverty status may represent material resources and financial hardships, while educational attainment may represent school quality and quantity. Given that poverty status captured more of the interactions than education alone, perhaps poverty status is more of the driver in these associations.

### Brain, stress, and disease

The general pattern of volumetric disparities observed in this study parallels those seen among other cardiovascular and neuroanatomical endpoints [36, 45]. The findings may therefore be relevant to health outcomes, given the burgeoning evidence suggesting the brain’s crucial role in the translation of stress to disease [4, 6]. For instance, the cardiovascular system is one of the most stress-sensitive physiological systems. Studies have found that increased medial PFC activity is associated with stress-evoked increases in heart rate [67, 68] and blood pressure [69]. Similar results have been found for the ACC and blood pressure [70]. Importantly, brain-mediated blood pressure rises in response to stress have been linked with accelerated atherosclerosis [71] and increased risk of myocardial infarction [72], suggesting the relations between stress and brain function may translate to important clinical implications for health. Further, a recent longitudinal study showed that amygdalar activity independently predicted cardiovascular disease outcomes [73], suggesting that amygdala function is one mechanism by which emotional stressors lead to adverse disease states. Given that neural function is related to its structure, longer-term brain changes that result from stress exposure may contribute to the acceleration of disease processes [74].

Several studies have linked stressful life experiences to structural changes in the brain. Controlled animal models have demonstrated that chronic stress exposure induces reductions in neuronal complexity and connectivity in cortical areas, including the medial PFC [75, 76]. In humans, lifetime stressors have been related in a dose-dependent fashion to hippocampus, amygdala, and medial PFC volumes [8, 77]. Stress-related structural alterations are notable considering that brain volume seems to be a robust indicator of the overall health of the brain and of brain-mediated physiological systems [78, 79]. Thus, race- and/or SES-related reductions in cortical and subcortical gray matter volume may contribute to the higher rates of disease observed in the low SES and AA population.

### Implications of examining brain volume

It is important to note several points when examining brain size. First, when considering the variability of gray matter volume in our sample, it is important to remember that reductions in brain volume are still within normal range of volumetric size, and not considered pathological. Relatedly, while statistical significance is informative, it is still unclear if the associations found between sociodemographic factors and brain volume confer biological importance. Second, it cannot be determined whether reduced gray matter density is a result of inferior neurodevelopment, subsequent increased neuronal and glial atrophy of a matured brain, or both. Research suggests that poverty and stressful life events during childhood are associated with suboptimal neurodevelopment [80]. This is particularly relevant in the context of racial health disparities, because compared to Whites, AAs are more likely to have encountered early life disadvantage [81]. Third, measures of brain volume are only one type of brain characteristic. When considering the functional implications of smaller brain volumes, it is crucial to note the influence of other brain outcomes, such as white matter hyperintensities, neural network connectivity, regional brain activity, as well as microstructural contributors like synaptic density.

### Limitations and strengths

Some limitations and future directions warrant discussion. First, the study’s cross-sectional design prohibits identification of temporal relations between sociodemographic factors and brain structure. Much of this manuscript assumes that SES-related factors lead to smaller brain volume, but this relation is likely bidirectional and longitudinal examinations are necessary to determine directionality of these associations. Second, our cumulative SES measure is limited. Although optimal assessment of SES is more nuanced than a dichotomy, HANDLS investigators based their initial area probability recruitment on a division of household income (as a function of household size) based on 125% of the 2004 federal poverty level. During the initial recruitment process, it was found that many HANDLS participants could not estimate their annual incomes, had no way to estimate their overall wealth, and were employed only sporadically. Therefore, HANDLS investigators used poverty status (a specific income level adjusted for household size) as their primary SES stratification variable. Additionally, our SES measure does not consider occupation, wealth, childhood SES, or variability of SES across the lifespan–variables that may independently contribute to brain volume. Third, our findings may not be generalizable to other races and ethnicities, and may underrepresent individuals living in upper middle classes. Fourth, while we utilized effect sizes as a secondary method of capturing meaningfully significant results, we did not correct our group contrasts for multiple comparisons due to concerns regarding type 2 error in this novel and largely exploratory study. Thus, the chance of type 1 error remains a concern. Fifth, while the literatures regarding 1) the negative effects of stress on brain volume and 2) higher rates of acute and chronic stress experienced by individuals with low SES and AAs are well-established, HANDLS did not collect an integrative lifespan measure of stress, and thus we are unable to confirm these relations in our sample. Future studies should seek to examine comprehensive, lifespan measures as mediators in the significant relations found here. Sixth, while we used a sophisticated multi-atlas segmentation method for delineation of our ROIs, certain relevant regions like the hypothalamus and brainstem (Fig 1) were not captured. Compared to research on the cortical and subcortical regions assessed here, the current literature on the impact of stress on the lower vegetative brain areas is limited. Future studies should therefore examine whether the hypothalamus and brainstem are also structurally vulnerable to the stressful life experiences encountered by those of lower SES and African Americans, particularly given that the hypothalamus is a target for stress hormones [82]. Seventh, we did not examine functional subdivisions of the PFC (e.g., dorsomedial PFC versus ventromedial PFC) or ACC (e.g., subgenual ACC versus pregenual ACC). While the various subdivisions of the PFC, for instance, are involved with the stress response [46], they seem to play differing roles in this process [83, 84]. The ventromedial PFC has been shown to lower the physiological stress response, in part by sending inhibitory projections to the subcortical structures [84, 85], while the dorsomedial PFC is associated with enhanced reactivity, potentially by sending excitatory projections to the subcortical regions [83, 86]. Given that these subdivisions respond differently to environmental threats and stressors, it would be prudent to examine how lifetime stress encountered by vulnerable populations differentially impacts these subdivisions. Finally, while not adjusting for ICV has its benefits (see Materials and Methods), variability in ROI volume may reflect variability in overall brain size.

This investigation also has several considerable strengths. The racially and economically diverse area probability sample of HANDLS provides considerable representation of historically underrepresented, disadvantaged groups. Additionally, although there is a burgeoning literature examining the SES-brain relation in children (e.g., [29]), our work adds to the relatively scant literature exploring the impact of socioeconomic and racial disparities on brain volume in adults.

## Conclusions

Our results suggest that relative to Whites with high SES, Whites with low SES and AAs are at a greater risk for smaller gray matter volumes in select stress-sensitive cortical brain regions. Our findings also suggest that on average, individuals with lower SES and AAs have smaller volumes in subcortical corticolimbic regions and the R orbital PFC and R ACC. These disparities are likely due to an aggregation of contextual stressors seen among self-identified AAs and those in disadvantaged socioeconomic positions. Smaller cerebral volumes in corticolimbic structures may contribute to less efficient stress perception and emotion regulation, possibly leading to a dysregulated corticolimbic circuit. This may, in part, contribute to the ostensible health disparities seen across sociodemographic groups. While speculative, these findings may demonstrate the brain’s role in mediating the translation of stress to disease. Clarification of the biological mechanisms involved in sociodemographic health disparities may provide guidance in efforts to alter disease trajectories among those at greatest risk.

## Supporting information

S1 Table Multiple regression analyses: Interaction of SES Indicators and race on gray matter volumes. R = Right; L = Left; PFC = prefrontal cortex; ACC = Anterior Cingulate Cortex; B = standardized regression coefficients; SE = standard error. *p < .05, **p < .01, ***p < .001. Full model: age, sex, race, SES indicator, SES indicator by race interaction.
(DOCX)

S2 Table Multiple regression analyses: Interaction of SES and race on total medial PFC and ACC, and control region. PFC = prefrontal cortex; ACC = anterior cingulate cortex; B = standardized regression coefficients; SE = standard error. **p < .01, ***p < .001. Full model: age, sex, race, SES, SES by race interaction.
(DOCX)
