# Supplementary material for: Sociodemographic disparities in corticolimbic structures
Source: PLoS One. 2019 May 9;14(5):e0216338. doi: 10.1371/journal.pone.0216338 (PMC6508895; doi:10.1371/journal.pone.0216338)
Supplement: S1 Table — R = Right; L = Left; PFC = prefrontal cortex; ACC = Anterior Cingulate Cortex; B = standardized regression coefficients; SE = standard error. *p < .05, **p < .01, ***p < .001. Full model: age, sex, race, SES indicator, SES indicator by race interaction. (DOCX) [file pone.0216338.s001.docx]

|  |  | **SES Interaction Term (All Ns = 200)** | | | | | |
| --- | --- | --- | --- | --- | --- | --- | --- |
| **Variable** |  | **Continuous Education** | | **Dichotomous Education** | | **Poverty Status** | |
|  |  | B | *SE* | B | *SE* | B | *SE* |
| R Medial PFC |  |  |  |  |  |  |  |
|  | Race | .05 | .33 | -.32*** | .07 | -.34*** | .08 |
|  | SES | .21** | .07 | -.21** | .08 | -.14 | .09 |
|  | SES x Race | -.30 | .33 | .14 | .08 | .23* | .10 |
| L Medial PFC |  |  |  |  |  |  |  |
|  | Race | .06 | .34 | -.22** | .07 | -.20* | .08 |
|  | SES | .13 | .07 | -.19* | .08 | -.14 | .09 |
|  | SES x Race | -.20 | .34 | .18* | .08 | .17 | .10 |
| R Orbital PFC |  |  |  |  |  |  |  |
|  | Race | -.55 | .32 | -.38 | .07*** | -.44*** | .08 |
|  | SES | .09 | .07 | -.08 | .07 | -.13 | .08 |
|  | SES x Race | .20 | .32 | .07 | .08 | .20* | .10 |
| L Orbital PFC |  |  |  |  |  |  |  |
|  | Race | -.31 | .33 | -.35*** | .07 | -.39*** | .058 |
|  | SES | .09 | .07 | -.09 | .08 | -.15 | .08 |
|  | SES x Race | .00 | .33 | .09 | .08 | .19 | .10 |
| R ACC |  |  |  |  |  |  |  |
|  | Race | .23 | .37 | -.25** | .08 | -.18* | .08 |
|  | SES | .08 | .08 | -.12 | .09 | .04 | .10 |
|  | SES x Race | -.41 | .37 | .15 | .09 | -.01 | .12 |
| L ACC |  |  |  |  |  |  |  |
|  | Race | .24 | .35 | -.39*** | .08 | -.15*** | .08 |
|  | SES | .15* | .07 | -.12 | .08 | -.05 | .09 |
|  | SES x Race | -.58 | .35 | .12 | .09 | .23* | .11 |
| R Hippocampus |  |  |  |  |  |  |  |
|  | Race | -.20 | .35 | -.26** | .07 | -.14 | .08 |
|  | SES | .08 | .08 | -.16 | .08 | -.08 | .09 |
|  | SES x Race | .02 | .36 | .17 | .09 | -.04 | .11 |
| L Hippocampus |  |  |  |  |  |  |  |
|  | Race | -.13 | .35 | -.29*** | .07 | -.20* | .08 |
|  | SES | .09 | .07 | -.17* | .08 | -.17 | .09 |
|  | SES x Race | -.09 | .35 | .16 | .09 | .02 | .11 |
| R Amygdala |  |  |  |  |  |  |  |
|  | Race | .30 | .36 | -.05 | .08 | .02 | .09 |
|  | SES | .11 | .08 | -.15 | .08 | -.11 | .09 |
|  | SES x Race | -.29 | .36 | .14 | .09 | .02 | .11 |
| L Amygdala |  |  |  |  |  |  |  |
|  | Race | .24 | .35 | -.23** | .07 | -.14 | .08 |
|  | SES | .13 | .07 | -.17* | .08 | -.17 | .09 |
|  | SES x Race | -.40 | .35 | .18* | .09 | .04 | .11 |
