# Supplementary material for: Sociodemographic disparities in corticolimbic structures
Source: PLoS One. 2019 May 9;14(5):e0216338. doi: 10.1371/journal.pone.0216338 (PMC6508895; doi:10.1371/journal.pone.0216338)
Supplement: S2 Table — PFC = prefrontal cortex; ACC = anterior cingulate cortex; B = standardized regression coefficients; SE = standard error. **p < .01, ***p < .001. Full model: age, sex, race, SES, SES by race interaction. (DOCX) [file pone.0216338.s002.docx]

| **Variable** |  | **B** | ***SE*** |
| --- | --- | --- | --- |
| Occipital Pole |  |  |  |
|  | Race | -.26** | .09 |
|  | SES | -.15 | .08 |
|  | SES x Race | .16 | .11 |
| Medial PFC |  |  |  |
|  | Race | -.34*** | .09 |
|  | SES | -.25** | .08 |
|  | SES x Race | .27* | .10 |
| ACC |  |  |  |
|  | Race | -.43*** | .09 |
|  | SES | -.13 | .09 |
|  | SES x Race | .24* | .11 |
